# Supplementary material for: Low Dosed Curcumin Combined with Visible Light Exposure Inhibits Renal Cell Carcinoma Metastatic Behavior in Vitros
Source: Cancers (Basel). 2020 Jan 28;12(2):302. doi: 10.3390/cancers12020302 (PMC7072295; doi:10.3390/cancers12020302)
Supplement: Supplementary file 1 [file cancers-12-00302-s001.pdf]

Figure S1. Whole Western Blots for figure 7.

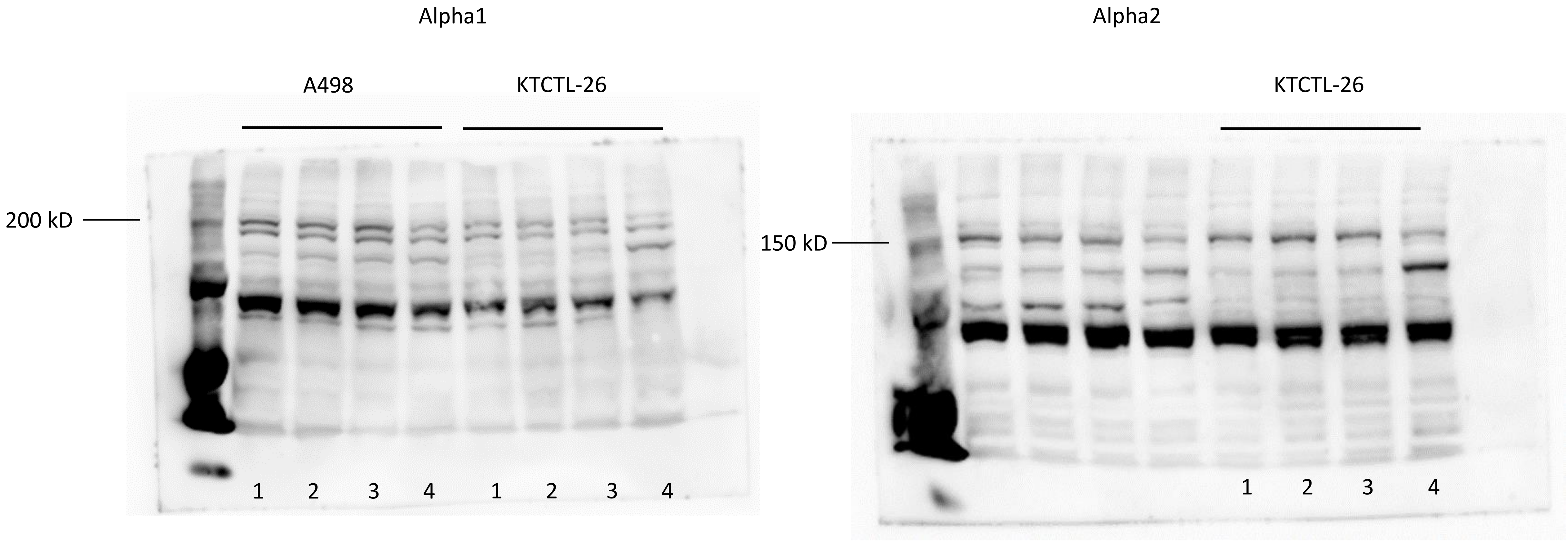

1=Control
2=Light
3=Curcumin
4=Curcumin^{Light}

Alpha3

A498 KTCTL-26

150 kD

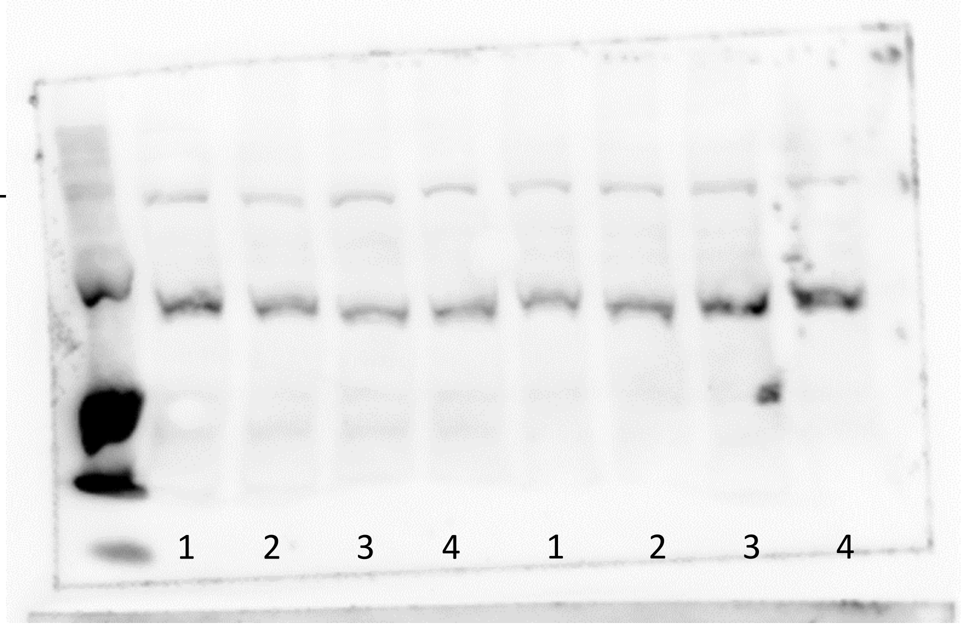

Caki1

150 kD

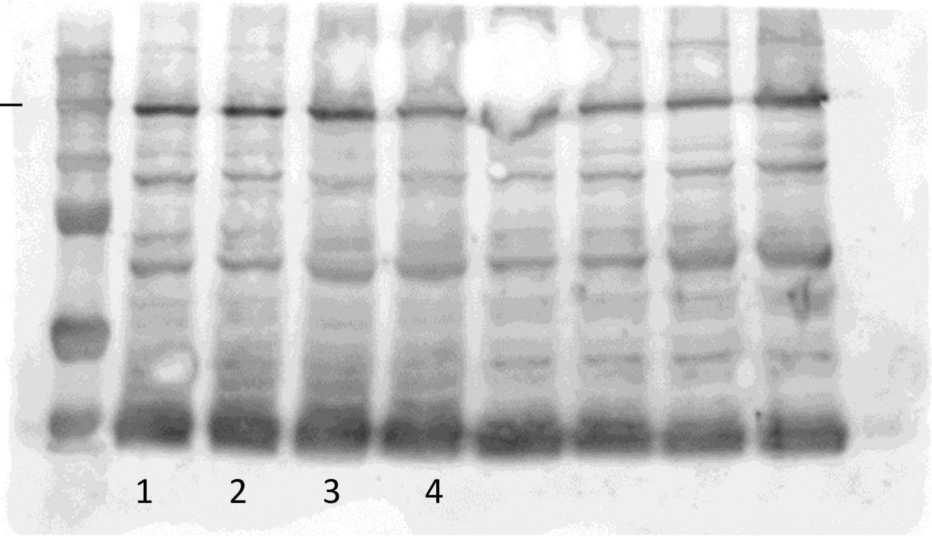

1=Control
2=Light
3=Curcumin
4=Curcumin^{Light}

Alpha5

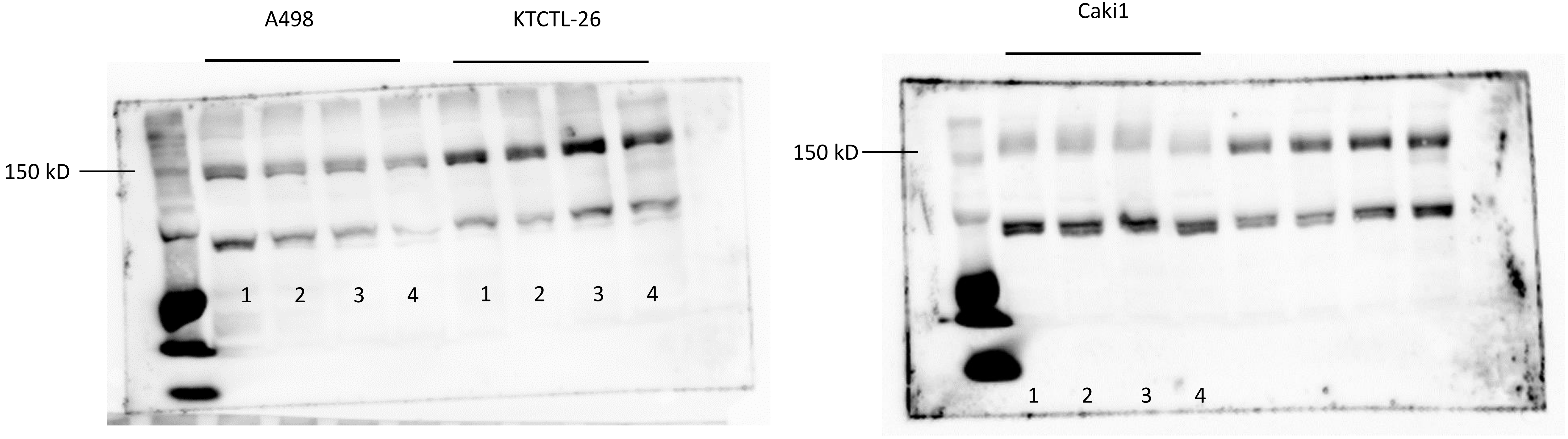

1=Control
2=Light
3=Curcumin
4=Curcumin^{Light}

Alpha6

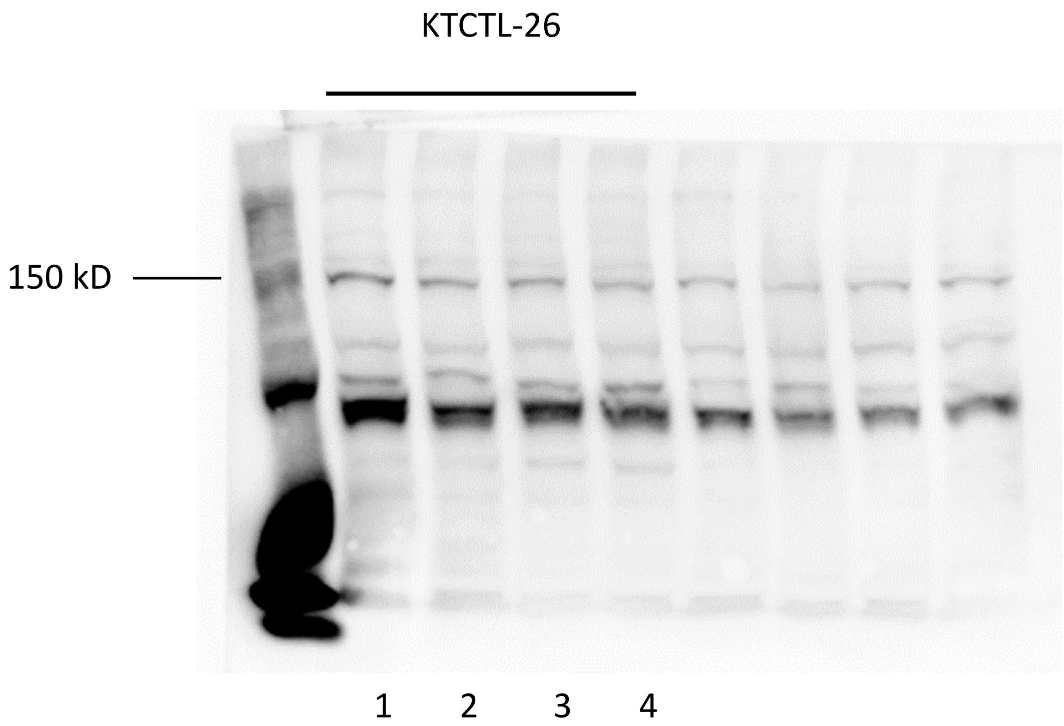

1=Control
2=Light
3=Curcumin
4=Curcumin[Light]

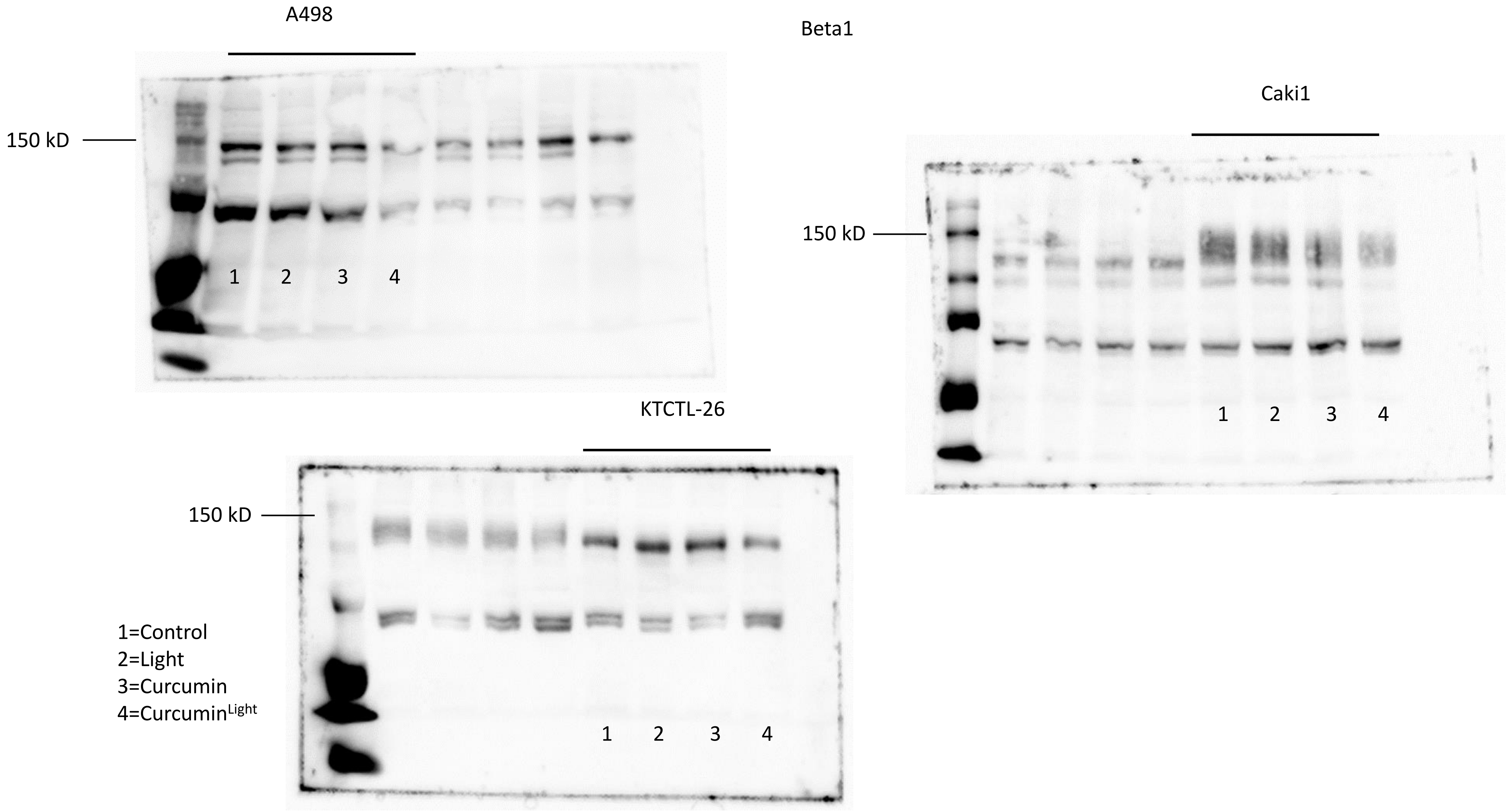
A498
Beta1
150 kD
1
2
3
4
Caki1
150 kD
1
2
3
4
KTCTL-26
150 kD
1=Control
2=Light
3=Curcumin
4=Curcumin^Light
1
2
3
4

Beta3

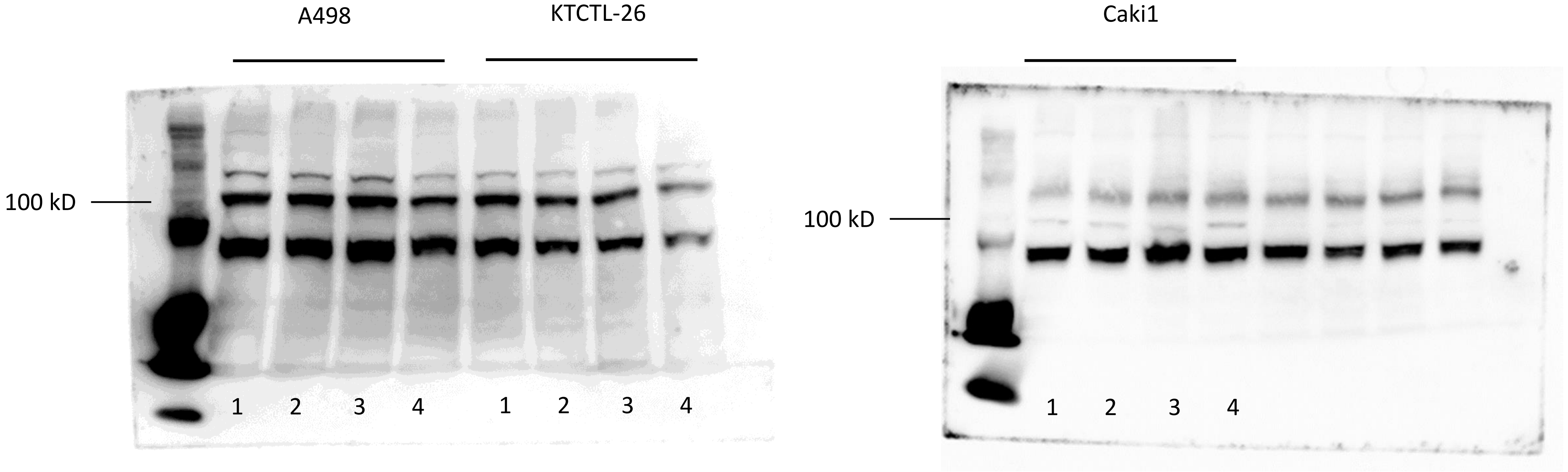

1=Control
2=Light
3=Curcumin
4=Curcumin^Light^

ILK

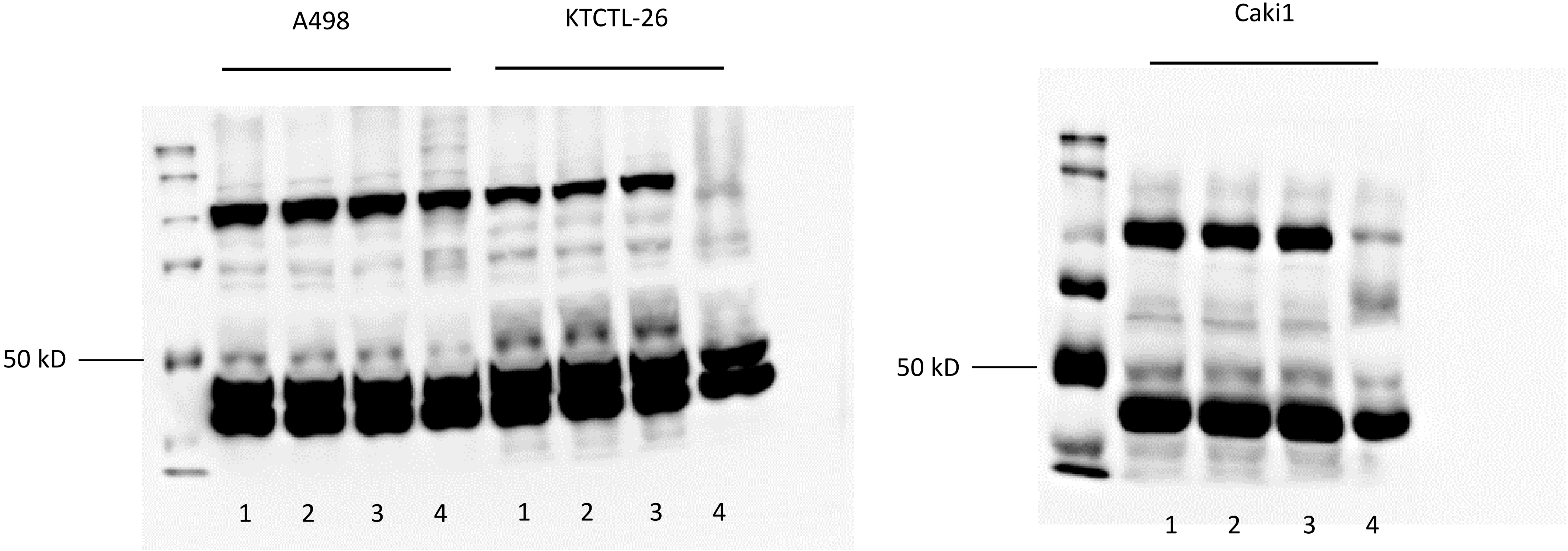

1=Control
2=Light
3=Curcumin
4=Curcumin^{Light}

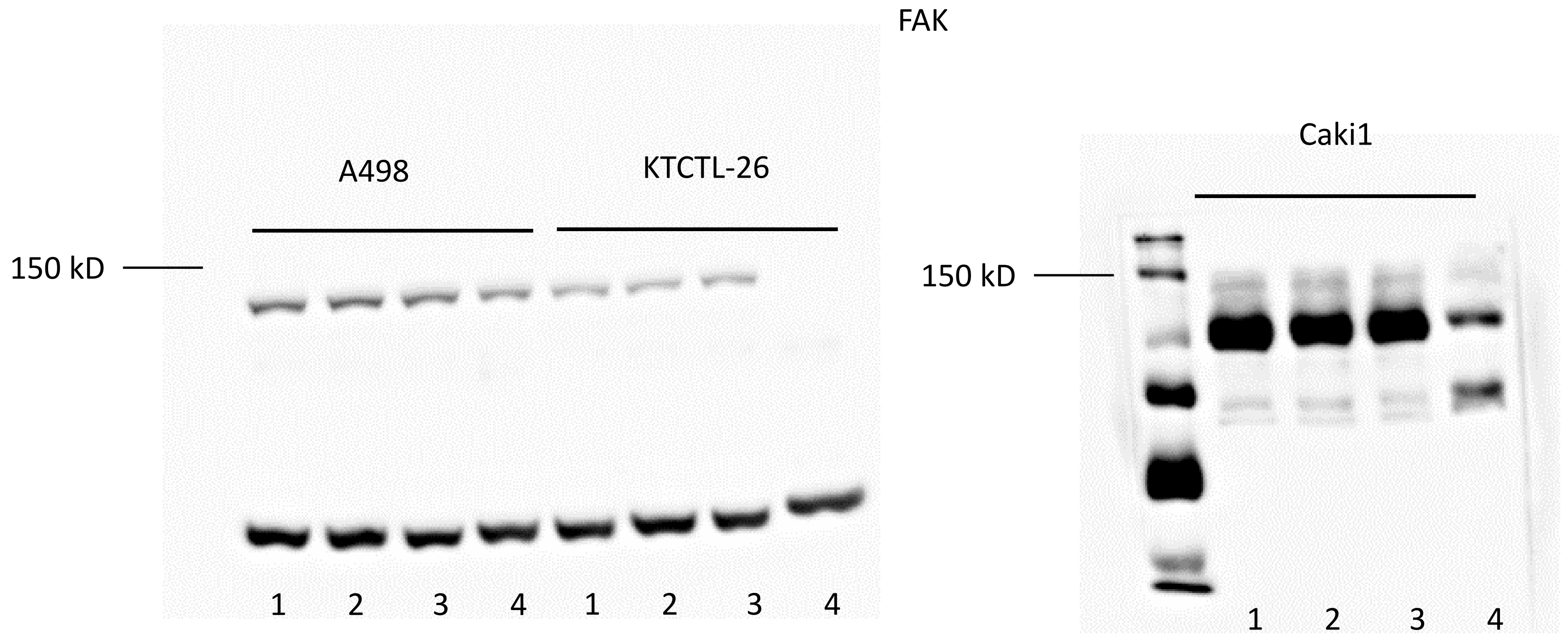

1=Control
2=Light
3=Curcumin
4=Curcumin^{Light}

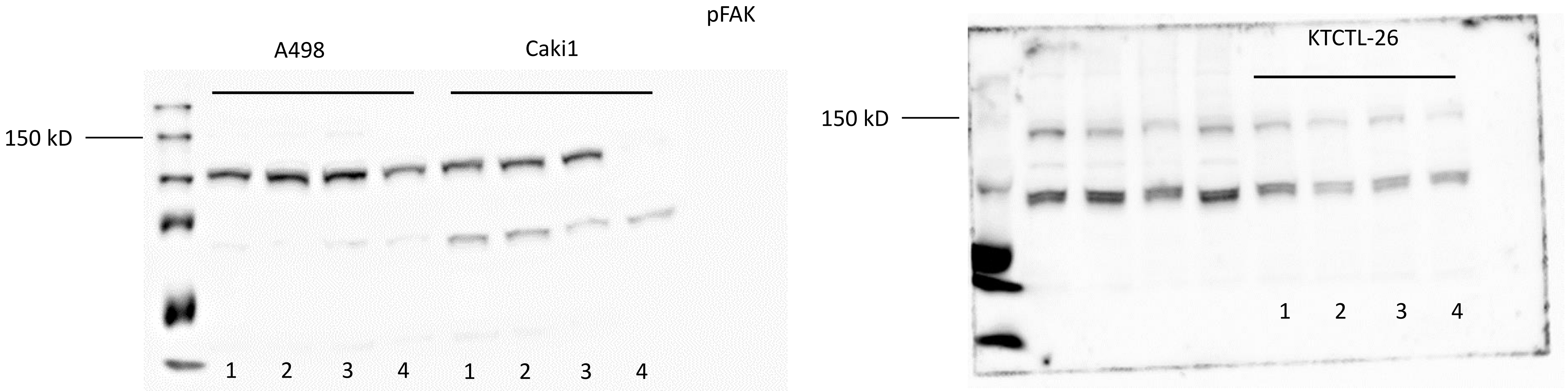

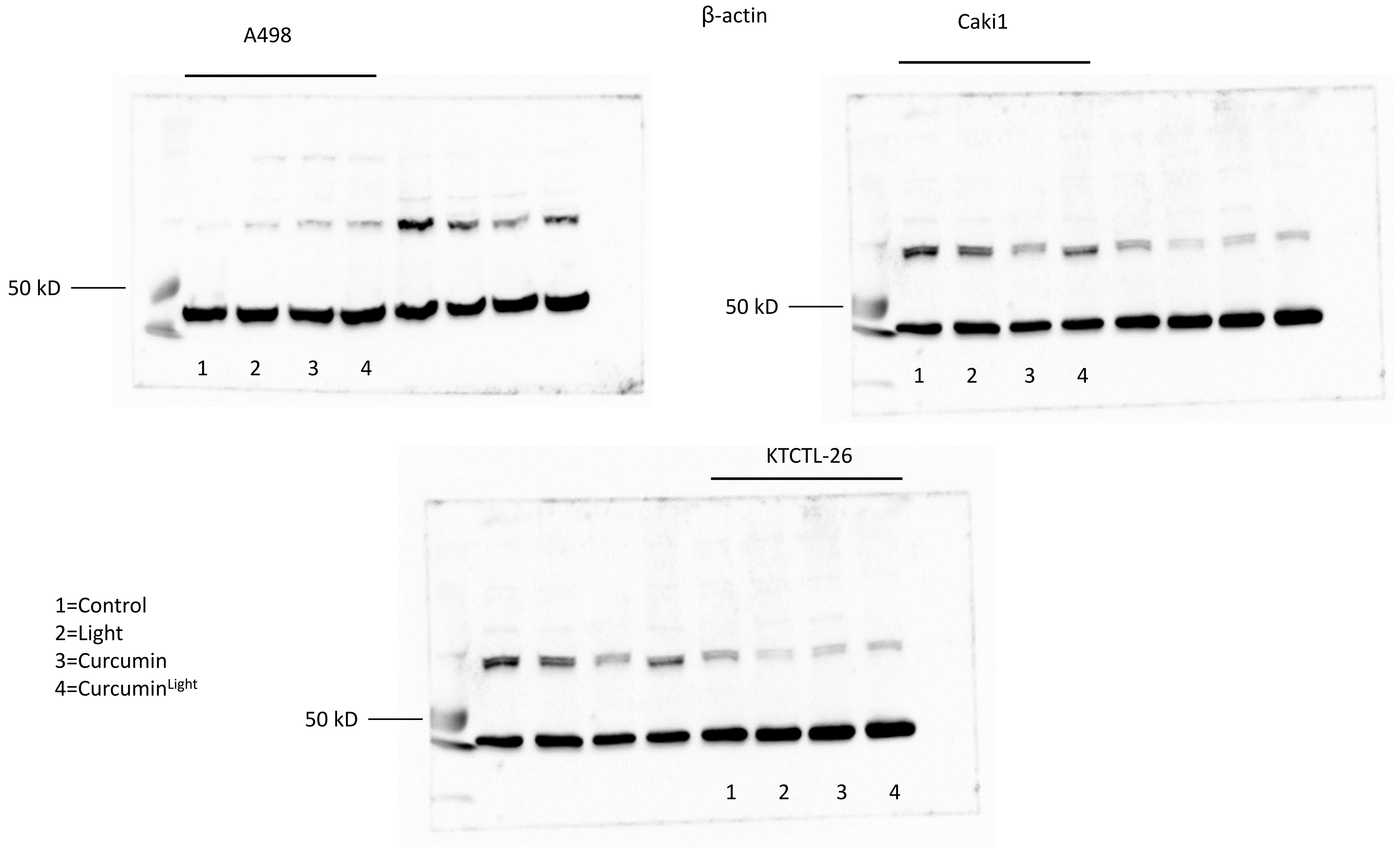
β-actin
A498
50 kD
1
2
3
4
Caki1
50 kD
1
2
3
4
KTCTL-26
50 kD
1
2
3
4
1=Control
2=Light
3=Curcumin
4=Curcumin^Light

Figure S2: Pixel density values for figure 8

A498	Control	Light	Curcumin	$Curcumin^{Light}$	p-values (control vs. $curcumin^{Light}$)
alpha1	96	91,1	99,7	61,4	0.0143
alpha2	n.d.				n.d.
alpha3	31	23,4	28,7	24,4	n.s.
alpha4	n.d.				n.d.
alpha5	94,6	64,6	65,3	56,6	n.s.
alpha6	n.d.				n.d.
beta1	92,7	63	56,3	33,5	0.0010
beta3	195,5	205,8	215,7	173,1	0.0462
beta4	n.d.				n.d.
ILK	88,8	81,2	81,7	51,2	0.0026
FAK	80,9	79,4	68,5	49,4	0.0265
pFAK	103,3	128	130,7	71,8	0.0008
beta-Aktin	181	170,4	172,5	187,8	

Caki1	Control	Light	Curcumin	$Curcumin^{Light}$	
alpha1	n.d.				n.d.
alpha2	n.d.				n.d.
alpha3	106,1	103,6	116,4	94,5	n.s.
alpha4	n.d.				n.d.
alpha5	50	49,4	44,9	35,2	0.0002
alpha6	n.d.				n.d.
beta1	122,7	120,7	87,8	59,5	0.0007
beta3	17,2	20,7	40,4	34	0.0210
beta4	n.d.				n.d.
ILK	100,3	94,2	84,8	45,9	0.0019
FAK	55,6	58,4	52,8	21,3	0.0066
pFAK	76,4	81,1	75,6	6,6	0.0008
beta-Aktin	215	228	199,2	206	

KTCTL-26	Control	Light	Curcumin	$Curcumin^{Light}$	
alpha1	69	66,6	64,5	38,9	0.0017
alpha2	64,1	80,2	68,3	51,6	0.0407
alpha3	20,4	22,1	24,9	21,3	n.s.
alpha4	n.d.				n.d.
alpha5	129,6	118,3	181,3	144,8	n.s.
alpha6	70	52,6	52,2	47,8	0.0398
beta1	99,8	125,5	125,4	69,5	0.0047
beta3	172,1	140,3	135,6	77	0.0008
beta4	n.d.				n.d.
ILK	124,8	133,6	143,7	57,2	0.0010
FAK	42,1	37,5	42,5	5,9	0.0008
pFAK	34,5	20,1	22,7	13,8	0.0018
beta-Aktin	188,7	193,3	206,1	222,4	

n.s.= not significant, n.d.= not done
